# Accuracy of Nurses’ Fall Prevention Interventions in Patients With Cognitive Impairment and Behavioral Symptoms

**DOI:** 10.1093/geroni/igaa057.753

**Published:** 2020-12-16

**Authors:** Deanna Gray-Miceli, Alison Kris

**Affiliations:** 1 Thomas Jefferson University, Philadelphia, Pennsylvania, United States; 2 Nursing, Fairfield, Connecticut, United States

## Abstract

Over 50 percent of older residents in nursing homes (NHs) fall each year. Many falls occur among NH patients with cognitive impairment and behavioral symptoms. Although NH nurses caring for patients who fall intervene to prevent fall recurrence, we know little about nurse’s perceptions of the most effective interventions for management of falls related to behavioral symptoms. The purpose of this qualitative study is to describe and analyze nurse’s perceptions of fall prevention interventions believed to be due to behavioral symptoms. This secondary analysis of existing qualitative data was conducted from a multi-site parent study in three NHs in the northeastern U.S. designed to test nurse’s knowledge of falls prevention and interventions. Forty-seven registered or licensed practical nurses, English speaking who were full or part time employees were recruited to participate. Most were female (n=46; 98.7%) with a median age of 49.5 years and ten years experience. A grounded theory approach was used to explore qualitative data (Glaser & Strauss, 1967) about nurse’s primary and secondary interventions. Qualitative data were collected in the form of responses to open ended questions from 47 nurses. The correctness of nurse interventions were independently evaluated, then validated among two independent experts. Cohen’s κ was used to determine if there was agreement between the experts’ judgement on correctness of the nurses’ intervention. Results indicated a high level agreement between expert evaluations (κ =.727-.760, p<.001). Emergent themes in the nurse interventions included: seeking outside help, confronting behavior, completing additional assessments, and patient reassurance.

